# Case report: Disseminated mucormycosis misdiagnosed as malignancy developed from allergic bronchopulmonary mycosis caused by *Rhizopus microsporus* following SARS-CoV-2 infection in a woman

**DOI:** 10.3389/fmed.2024.1394500

**Published:** 2024-06-26

**Authors:** Chengying Kong, Laibin Zong, Shunxian Ji, Yangxiang Liu, Miaomiao Li

**Affiliations:** ^1^Department of Respiratory and Critical Care Medicine, The Fourth Affiliated Hospital, Zhejiang University School of Medicine, Yiwu, Zhejiang, China; ^2^Department of Clinical Laboratory, The Fourth Affiliated Hospital of Zhejiang University School of Medicine, Yiwu, Zhejiang, China; ^3^Department of Pathology, The Fourth Affiliated Hospital, Zhejiang University, School of Medicine, Yiwu, Zhejiang, China

**Keywords:** mucormycosis, pulmonary mucormycosis, *Rhizopus* spp., allergic bronchopulmonary mycosis (ABPM), SARS-CoV-2, case report

## Abstract

Mucormycosis has become more prevalent during the COVID-19 pandemic and is associated with a high mortality rate. However, concurrent host allergic reactions, invasive pulmonary mucormycosis, and disseminated mucormycosis are rarely reported. Herein, we describe a case of disseminated mucormycosis initially misdiagnosed as a malignancy that developed from allergic bronchopulmonary mycosis caused by *Rhizopus microsporus* in a woman with post-SARS-CoV-2 infection. The previously healthy patient presented with a sizeable mass in the right middle lobe and multiple lesions across the lungs, brain, spleen, kidneys, pancreas, and subcutaneous tissue 6 months after SARS-CoV-2 infection, mimicking an extensive metastatic malignancy. Eosinophilia, elevated total plasma immunoglobulin E, and significant eosinophilic lung tissue infiltration were observed. *Rhizopus microsporus* was isolated from subcutaneous tissue, and hyphae were detected in the lung tissue. Sequential amphotericin B liposomes followed by isavuconazole antifungal therapy combined with systemic corticosteroids improved symptoms, significantly reduced the sizes of pulmonary lesions, and reduced eosinophil count. However, it failed to halt the overall progression of the disease, and the patient died. The absence of asthma-like symptoms and delayed recognition of invasive fungal infection signs contributed to poorer outcomes, highlighting the need for a thorough post-COVID-19 follow-up.

## 1 Introduction

Mucormycosis is a fungal infection caused by fungi within the *Mucorales* order. It is an emerging disease characterized by high morbidity and mortality and poses challenges in diagnosis (1). The mortality rate varies based on the site of infection: 76% for pulmonary infections, 96% for disseminated disease, and 100% for general dissemination (2). Individuals with immune deficiencies are susceptible, and reports of COVID-19-associated mucormycosis (CAM) have surged since early 2021 (3). *Rhizopus* spp. are the dominant causative agents of mucormycosis worldwide (1, 4). *Rhizopus* belongs to the *Mucoraceae* family (5) and is commonly found in many environments. *R. arrhizus* is the most common agent of mucormycosis globally compared to other species.

Allergic bronchopulmonary mycosis (ABPM) is a hypersensitivity-mediated disease of the lower airways caused by environmental fungi, with *Aspergillus fumigatus* being the most common, defined as allergic bronchopulmonary aspergillosis (ABPA). Severe asthma is an important feature of ABPA (6). Nevertheless, 70% of cases of ABPM due to fungi other than *Aspergillus* do not involve asthma (7). *Rhizopus* spp. are rarely reported as causative agents of ABPM (7).

Here, we describe a case of disseminated mucormycosis misdiagnosed as a malignancy, which developed from ABPM caused by *Rhizopus microsporus* in a woman post-SARS-CoV-2 infection. We isolated *R. microsporus* from her subcutaneous tissue without any trauma and detected hyphae and eosinophilic infiltration in her lung tissue. *R. microsporus* belongs to the *Rhizopus* genus, which is relatively uncommon compared with other species (5).

## 2 Case description

A previously healthy 60-year-old housewife who contracted SARS-CoV-2 infection in December 2022 was admitted to our hospital on June 22, 2023. At admission, the patient reported prolonged fever for nearly 1 month, accompanied by cough, chest stress, progressive fatigue, and loss of appetite. Her previous suspected diagnosis of lung cancer with metastasis was based on her imaging manifestations, including a significant mass in the right middle lobe causing bronchus occlusion, atelectasis, nodules in the lower right lobe, multiple lymph node enlargements in the right hilum and mediastinum, and right pleural effusion on her enhanced chest computed tomography (CT). Additionally, a neoplasm obstructing the right middle bronchus was identified under tracheoscopy. However, no malignant cells were found in her pleural fluid, tracheoscopic biopsy of the neoplasm obstructing the right middle bronchus, or pulmonary biopsy of the nodule in the lower right lobe.

**Figure 1:**
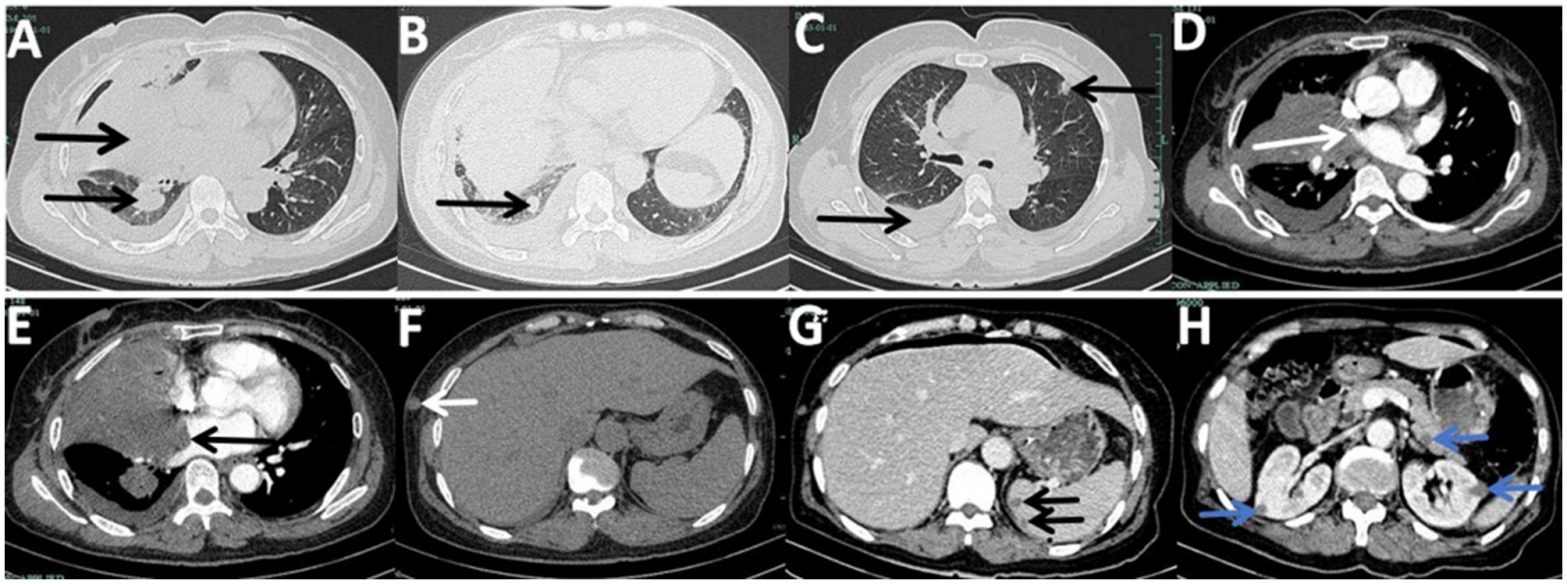
Enhanced chest computed tomography (CT) and enhanced abdominal CT at admission captured layers with lesions, although not all lesions are depicted. **(A)** Mass in the right middle lobe with bronchus occlusion, atelectasis, and a nodule in the lower right lobe, previously punctured. **(B)** New lower right lobe nodule compared to her first chest CT from the previous hospital. **(C)** Right-sided pleural effusion and a new lesion in the left upper lobe. **(D)** Interruption of the right upper pulmonary vein by the right middle lobe. **(E)** Invasion of the left atrium and mural thrombosis by the right middle lobe mass. **(F)** Nodule in the right chest wall. **(G)** Double nodules in the spleen. **(H)** Wedge-shaped nodules in her kidneys and a nodule in the pancreas.

**Figure 2:**
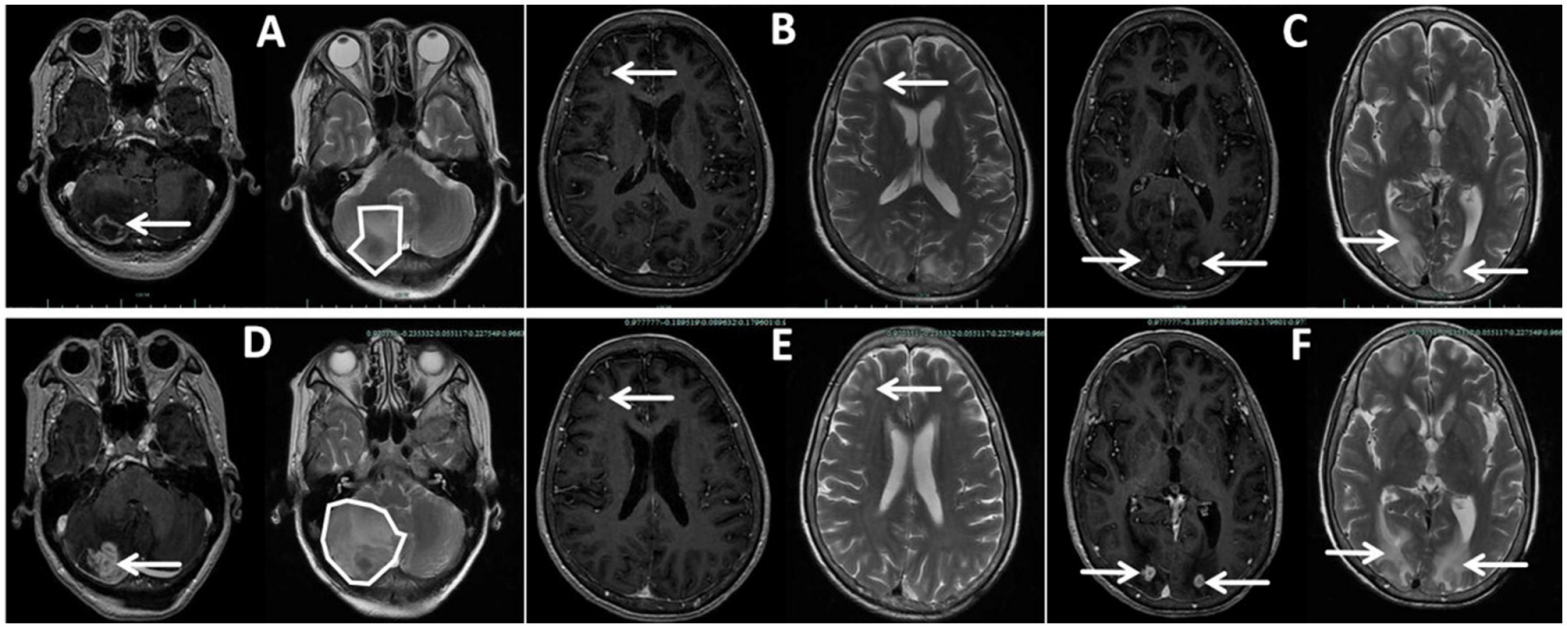
Enhanced brain magnetic resonance imaging (MRI) of the patient taken at admission **(A**–**C)** and Day 65 **(D**–**F)**, showing some but not all lesions. **(A, D)** Lesion in the right cerebellar hemispheres, larger on Day 65 than at admission, with wider brain edema and a left-shifted midline with the fourth ventricle more compressed. **(B, E)** Lesion in the right frontal lobe, smaller on Day 65 than at admission. **(C, F)** Double lesions of the occipital lobe, larger on Day 65 than at admission.

New nodules were observed on her left forearm, left thigh, and left lower back at admission, which gradually increased in size over a month. Peripheral blood analysis showed eosinophilia of 2,040-3,290/μl (normal range: 20–520/μl) and elevated total plasma IgE of >2,500 IU/ml (normal range: < 100 IU/ml), indicating host allergic reaction. Additional nodules were found in the lower right lobe, and the right middle lobe mass invaded her right pulmonary veins and left atrium on enhanced chest CT at 1 month (Figures 1A–E). Enhanced chest and abdominal CT revealed multiple nodules in her right chest wall, kidneys, spleen, and pancreas (Figures 1F–H). Brain-enhanced magnetic resonance imaging (MRI) at admission showed ring-enhancing lesions in the right cerebellar hemisphere, right frontal lobe, and bilateral occipital cutaneous medullary junction, as well as abnormal signals in the right frontal lobe with meningeal enhancement (Figures 2A–C).

## 3 Diagnostic assessment

**Figure 3:**
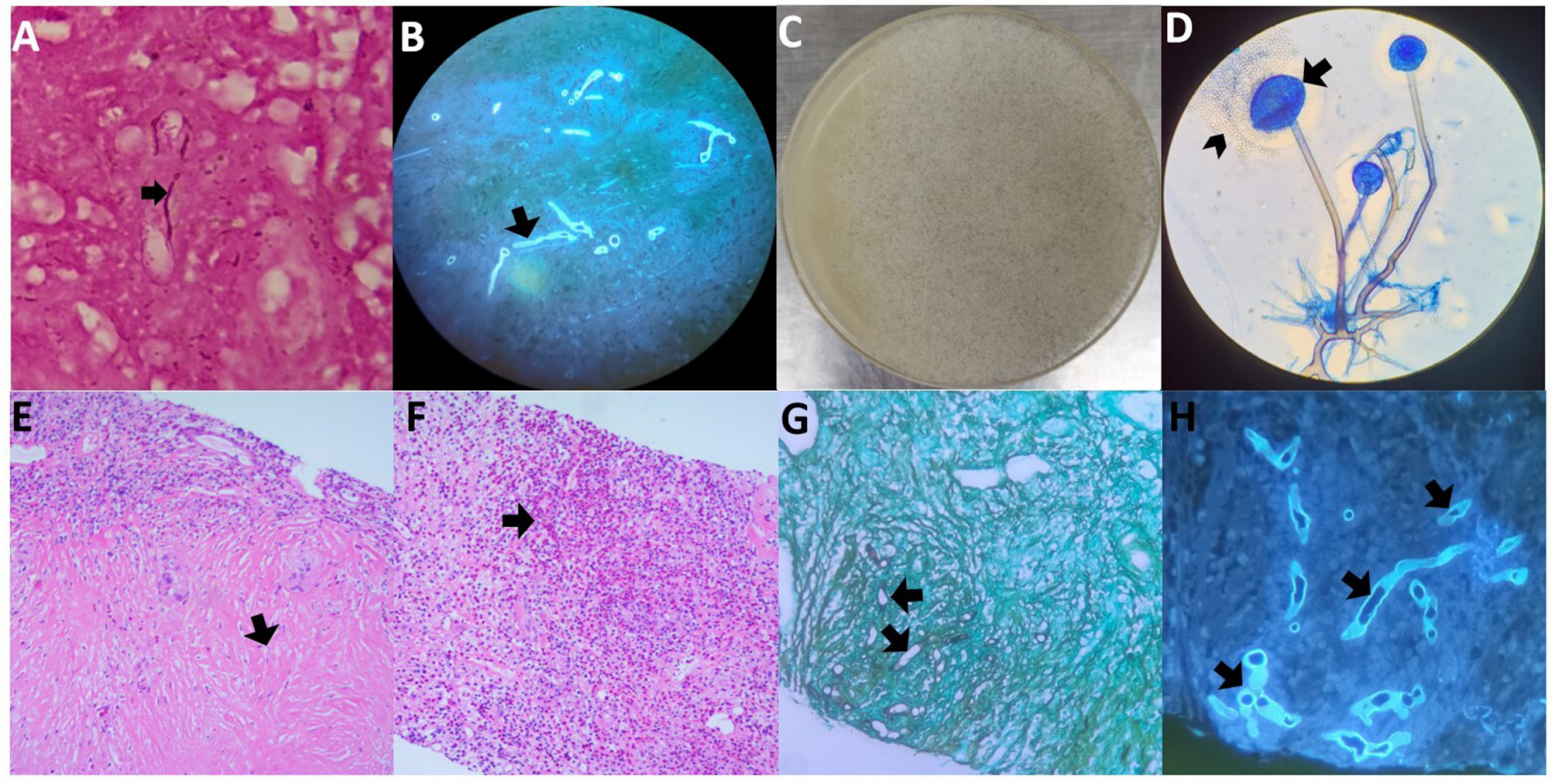
Histopathology and microbiology findings from both biopsy specimens. **(A)** Wide, non-septate hyphae in the Gram staining of the left forearm nodule specimen. **(B)** Wide, non-septate hyphae in the fungal fluorescence of the left forearm nodule specimen. **(C)** Cultured subcutaneous nodule was cultured on potato glucose agar showing *Rhizopus microspora* growth at 35°C for four days, with a moderately floccose mycelium colony. **(D)** Lactophenol cotton blue staining of the isolated strain, and its globose small sporangium (arrows) is dissolving and releasing oval or irregular sporangiospore (arrowheads). **(E, F)** Hematoxylin and eosin (H&E) staining of the right lower lung puncture specimen, **(E)** indicates chronic collagen fibrous hyperplasia, and **(F)** indicates a huge amount of eosinophilic infiltration. **(G)** Wide, non-septate hyphae resembling those in subcutaneous tissue with periodic-acid silver methenamine staining of the specimen from the right lower lung puncture. **(H)** Wide, non-septate hyphae resembling those in subcutaneous tissue with fungal fluorescence of the specimen of the right lower lung puncture.

A surgical biopsy of the subcutaneous nodule in the left forearm was performed. Wide, non-septate hyphae were found using Gram staining (Figure 3A) and fungal fluorescence (Figure 3B) of the left forearm nodule specimen. Further, 144,129 reads of *R. microsporus* were detected by metagenomics next-generation sequencing in the specimen with relative abundance of 98.43% (the sequencing data reported in this study was archived in the Sequence Read Archive (SRA) under accession number PRJNA1106391). Part of the tissue from the same specimen was cultured on potato glucose agar at 35°C for 4 days (Figures 3C, D); extracted DNA from the cultured strains was sequenced with internal transcribed spacer sequences, and the DNA sequence was identified as *R. microsporus* by comparing with the Basic Local Alignment Search Tool (the sequencing data reported in this study was archived in GenBank under accession number SUB14426961 20240406001 PP766957). Disseminated subcutaneous mucormycosis was confirmed. Furthermore, lung puncture specimen analysis revealed wide, non-septate hyphae with chronic collagen fibrous hyperplasia, accompanied by a significant presence of eosinophilic infiltration and a few multinuclear giant cell reactions, indicating invasive pulmonary mucormycosis overlapping with lung tissue allergic reaction (Figures 3E–H).

These findings confirmed the diagnosis of invasive pulmonary mucormycosis overlapping with ABPM caused by *R. microsporus* and disseminated subcutaneous mucormycosis. The multifocal disease involving the central nervous system, spleen, kidneys, and pancreas was suspected to be disseminated mucormycosis.

## 4 Details on the therapeutic intervention

**Figure 4:**
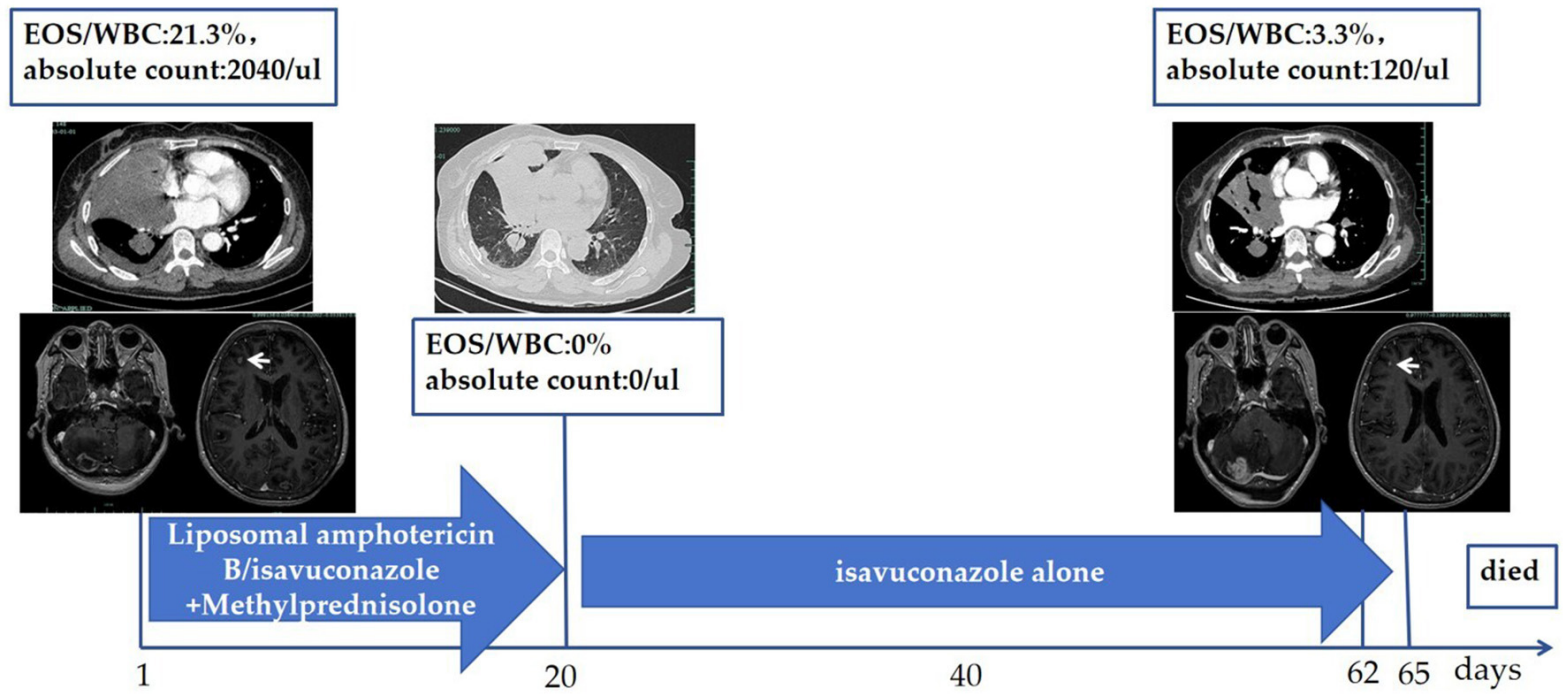
Treatment course. Liposomal amphotericin B (10 mg/kg, QD) administered intravenously from Day 1 to Day 6, followed by isavuconazole (200 mg Q8H for the first 48 h intravenously, then 200 mg QD orally) from Day 7. Simultaneously, methylprednisolone was administrated with 40 mg/day (body weight of 53 kg, prednisone equivalent of 1 mg/kg) from Day 1, reduced to 32 mg/day from Day 8, and gradually tapered. The patient's peak body temperature declined on Day 2 and returned to normal on Day 4. She suffered from gastric perforation on Day 20, leading to the discontinuation of methylprednisolone. Reduction in pulmonary lesions and decreased eosinophils were observed. Hemoptysis, multiple pulmonary embolisms, and intracranial hypertension occurred on Days 62–65, resulting in the patient's death. WBC, white blood cells; EOS, Eosinophils; QD, once daily; Q8H, every 8 h.

Debridement was not suitable for this patient due to the disseminated lesions. The treatment course (Figure 4) was divided into two phases due to the complication of gastric perforation.

The first phase was composed of antifungal agents and systemic corticosteroids. For the antifungal agents, liposomal amphotericin B was administrated intravenously at a dosage of 10 mg/kg/day from Day 1 to Day 6, for a total of 6 days. Then, isavuconazole was administrated intravenously with a loading dose of 200 mg every 8 h for the first 48 h beginning on Day 7, and then converted to an oral isavuconazole capsule with a dosage of 200 mg per day until Day 20, for a total of 14 days. For systemic corticosteroids, methylprednisolone was administrated intravenously at a dosage of 40 mg/day (patient's body weight was 53 kg, prednisone equivalent of 1 mg/kg) from Day 1, reduced to 32 mg/day orally beginning at Day 8, and gradually tapered until Day 20. The patient's symptoms were alleviated, and she was discharged on Day 8 to continue with the oral isavuconazole capsules at home. A chest CT on Day 20 showed complete absorption of her right pleural effusion and a reduction in the size of the pulmonary lesions.

On Day 20, the patient experienced a sudden stomach ache, leading to a diagnosis of gastric perforation, which necessitated surgical repair and caused the treatment process to transition into the second phase. Methylprednisolone was discontinued due to the complication of gastric perforation, while isavuconazole capsules were continued at a dosage of 200 mg per day post-operation.

## 5 Follow-up and outcomes

On Day 62, the patient developed hemoptysis due to cavity formation in the right middle lobe, and multiple pulmonary embolisms were detected. On Day 65, she developed nausea and vomiting, which was exacerbated by changing positions. A brain-enhanced MRI (Figures 2D–F) revealed a reduced frontal lobe but increased size in other areas with severe edema. The patient declined further treatment due to her deteriorating condition and economic pressures associated with the cost of antifungal agents. She died 2 months after discharge due to intracranial hypertension caused by mucormycosis of the central nervous system.

## 6 Discussion

Mucormycosis is an emerging disease that has surged since early 2021 due to the COVID-19 pandemic. The most frequently reported causative agents of mucormycosis have been *Rhizopus* spp. (1), with *R. arrhizus* being the most common species, mainly associated with rhino-orbital cerebral mucormycosis (4, 8). However, concurrent host allergic reactions, invasive pulmonary mucormycosis, and disseminated mucormycosis have been rarely reported. Further, *Rhizopus* spp. are rarely seen in ABPM cases; Chowdhary et al. reported only two in 143 ABPM cases (7). Moreover, *R. microsporus* is relatively uncommon compared to other species of *Rhizopus* and is characterized by a moderately floccose mycelium colony; globose small sporangium, oval, or irregular sporangiospores that are 4–11 μm in diameter; and has been found distributed in soil, on fowl's skin scrapings, and moldy lumber (5).

Disseminated mucormycosis developed from ABPM overlaps with pulmonary mucormycosis caused by *R. microsporus* based on the following findings. Firstly, *R. microsporus* was isolated from the patient's subcutaneous tissue in high abundance, indicating *R. microsporus* was almost the only microorganism present. Secondly, the patient presented with eosinophilia and increased plasma IgE, indicating a host allergic reaction. Thirdly, significant eosinophilic infiltration and a few multinuclear giant cell reactions were observed in her lung tissue, suggesting lung tissue allergic reaction. Fourthly, several wide, non-septate hyphae similar to those in the subcutaneous tissue were detected in the necrotic lung tissue, verifying concurrent invasive pulmonary mucormycosis caused by *R. microsporus*. Fifthly, multiple subcutaneous nodules appeared subsequent to the eosinophilia and lung lesions during the progression of the disease, suggesting that disseminated subcutaneous mucormycosis was due to ABPM overlapping with pulmonary mucormycosis. Finally, the right pleural effusion was absorbed, pulmonary lesions were reduced, and certain extra-pulmonary lesions were reduced after antifungal therapy combined with systemic corticosteroids. It is reasonable to presume that multifocal disease involving the central nervous system, spleen, kidneys, and pancreas was due to disseminated mucormycosis.

*Rhizopus* spp. are opportunistic pathogens. Immunocompromised patients, such as those with uncontrolled diabetes, hematologic malignancy, or those taking immunosuppressive agents and chemotherapy, are susceptible (1, 9). Cutaneous or subcutaneous mucormycosis is generally observed in patients with trauma (1). Recommendations for the diagnosis and management of CAM (4) and COVID-19-associated pulmonary mucormycosis (CAPM) (10) have been published. CAPM refers to pulmonary mucormycosis occurring within 3 months of a COVID-19 diagnosis (10). Our patient's condition was unique as she had a transient SARS-CoV-2 infection 6 months prior to the onset of disease. In addition, this patient alleged that she was healthy previously, denied any chronic disease; screening tests for diabetes, hematopoietic malignancies, and HIV were negative, and levels of TBNK lymphocyte subsets, immunoglobulins, and complements were within normal range. Thus, she appeared to have no risk factors. However, SARS-CoV-2 infection may induce dedifferentiation of multiciliate cells and impair mucociliary clearance (11). We hypothesize that the impaired ciliary clearance created opportunities for sporangiospore of *R. microsporus* to colonize, germinate, trigger host allergic reactions, invade lung tissues and their vessels, and ultimately lead to hematogenous dissemination.

For mucormycosis patients, antifungal agents combined with early debridement are the keys to prolonged survival (10). Control of the underlying disease is also important (12). Recommended antifungal agents include liposomal amphotericin B, posaconazole, and isavuconazole.

However, there are no recommendations for the co-occurrence of host allergic reactions, invasive pulmonary mucormycosis, and disseminated subcutaneous mucormycosis thus far. Zhou et al. reported a successful case of invasive pulmonary mucormycosis overlapping with allergic diseases in a patient who was treated with systemic corticosteroids combined with posaconazole antifungal therapy (13), and the localized pulmonary lesion was ultimately removed. For our patient, sequential amphotericin B liposome followed by isavuconazole antifungal therapy combined with systemic corticosteroids rapidly alleviated symptoms, reduced the size of the lung lesions, and suppressed peripheral blood eosinophilia. However, it did not stop the progression of the majority of the extra-pulmonary lesions. The patient died of intracranial hypertension due to mucormycosis of the central nervous system. Systemic corticosteroids may be very helpful to decrease eosinophils and shrink lung lesions. A lost opportunity for early debridement may be disastrous to patients with disseminated mucormycosis. Thus, for patients with a history of SARS-CoV-2 infection, thorough post-COVID-19 follow-up is key to early detection, diagnosis, and treatment for mucormycosis.

This study has several limitations. Firstly, the absence of histopathology and microbiology findings in the central nervous system, spleen, or kidneys could be due to the potential harm caused by multiple organ biopsies. We assumed that multifocal diseases should be considered as mucormycosis based on disease monism. Secondly, we speculated that this patient had ABPM caused by *R. microsporus* based on clinical evidence and histopathology. Direct evidence of allergy to Mucor-specific IgE was absent because of experimental limitations. Thirdly, our patient lacked a follow-up visit after transient SARS-CoV-2 infection; therefore, we could not determine the true onset of the disease or the true risk. The patient had no history of any other disease, suggesting that SARS-CoV-2 infection alone may be a risk factor for ciliary clearance damage.

This was a rare case of disseminated mucormycosis that developed from ABPM and pulmonary mucormycosis caused by *R. microsporus* in a post-SARS-CoV-2 infection patient. The lack of asthma-like symptoms and signs of invasive fungal infections led to delayed diagnosis, resulting in poor outcomes. This case emphasizes the potential increase of ABPM incidence during or after the COVID-19 pandemic, highlighting the importance of closely monitoring patients with SARS-CoV-2 infection, even if they are asymptomatic, to prevent adverse outcomes.

## 7 Patient perspective

The patient had visited three hospitals seeking help and underwent three tissue biopsies. She and her family gained a certain understanding of mucormycosis. They were initially pleased with the effectiveness of the treatment but became frustrated when the long course of expensive drugs failed to prevent the progression of the extra-pulmonary disease. Her family also recognized the importance of close follow-up for COVID-19 patients.

## Data availability statement

The original contributions presented in this study are included in the article. Further inquiries can be directed to the corresponding author.

## Ethics statement

This study was conducted in accordance with the Declaration of Helsinki and was reviewed and approved by the Research Ethics Committee of the Fourth Affiliated Hospital of Zhejiang University School of Medicine (K2024032). The patient provided written informed consent for the publication of clinical information, including CTs, MRIs, and microbiological and pathological images. Written informed consent was obtained from the participant/patient(s) for the publication of this case report.

## Author contributions

CK: Conceptualization, Methodology, Writing – original draft, Writing – review & editing. LZ: Writing – review & editing. SJ: Writing – review & editing. YL: Supervision, Writing – review & editing. ML: Supervision, Writing – review & editing.

## References

[1] Cornely OA Alastruey-Izquierdo A Arenz D Chen SCA Dannaoui E Hochhegger B. Global guideline for the diagnosis and management of mucormycosis: an initiative of the European Confederation of Medical Mycology in cooperation with the Mycoses Study Group Education and Research Consortium. Lancet Infect Dis. (2019) 19:e405–21. 10.1016/S1473-3099(19)30312-3 31699664 PMC8559573

[2] Roden MM Zaoutis TE Buchanan WL Knudsen TA Sarkisova TA Schaufele RL. Epidemiology and outcome of zygomycosis: a review of 929 reported cases. Clin Infect Dis. (2005) 41:634–53. 10.1086/432579 16080086

[3] Hoenigl M Seidel D Carvalho A Rudramurthy SM Arastehfar A Gangneux JP. The emergence of COVID-19 associated mucormycosis: a review of cases from 18 countries. Lancet Microbe. (2022) 3:e543–52. 10.1016/S2666-5247(21)00237-8 35098179 PMC8789240

[4] Rudramurthy SM Hoenigl M Meis JF Cornely OA Muthu V Gangneux JP. ECMM/ISHAM recommendations for clinical management of COVID-19 associated mucormycosis in low- and middle-income countries. Mycoses. (2021) 64:1028–37. 10.1111/myc.13335 34133816 PMC8447004

[5] Ribes JA Vanover-Sams CL Baker DJ. Zygomycetes in human disease. Clin Microbiol Rev. (2000) 13:236–301. 10.1128/CMR.13.2.236 10756000 PMC100153

[6] Rodrigues J Caruthers C Azmeh R Dykewicz MS Slavin RG Knutsen AP. The spectrum of allergic fungal diseases of the upper and lower airways. Expert Rev Clin Immunol. (2016) 12:531–50. 10.1586/1744666X.2016.1142874 26776889

[7] Chowdhary A Agarwal K Kathuria S Gaur SN Randhawa HS Meis JF. Allergic bronchopulmonary mycosis due to fungi other than *Aspergillus*: a global overview. Crit Rev Microbiol. (2014) 40:30–48. 10.3109/1040841X.2012.754401 23383677

[8] Morales-Franco B Nava-Villalba M Medina-Guerrero EO Sánchez-Nuño YA Davila-Villa P Anaya-Ambriz EJ. Host-pathogen molecular factors contribute to the pathogenesis of *Rhizopus* spp. in diabetes mellitus. Curr Trop Med Rep. (2021) 8:6–17. 10.1007/s40475-020-00222-1 33500877 PMC7819772

[9] Prakash H Chakrabarti A. Global epidemiology of mucormycosis. J Fungi. (2019) 5:26. 10.3390/jof5010026 30901907 PMC6462913

[10] Muthu V Agarwal R Patel A Kathirvel S Abraham OC Aggarwal AN. Definition, diagnosis, and management of COVID-19-associated pulmonary mucormycosis: Delphi consensus statement from the Fungal Infection Study Forum and Academy of Pulmonary Sciences, India. Lancet Infect Dis. (2022) 22:e240–53. 10.1016/S1473-3099(22)00124-4 35390293 PMC8979562

[11] Robinot R Hubert M de Melo GD Lazarini F Bruel T Smith N. SARS-CoV-2 infection induces the dedifferentiation of multiciliated cells and impairs mucociliary clearance. Nat Commun. (2021) 12:4354. 10.1038/s41467-021-24521-x 34272374 PMC8285531

[12] Thakur Rai N Misra M Misra S Misra S Shukla DK Singh AK. Insulin and early debridement keys to survival in-COVID 19 associated mucormycosis patients (CAM) - an experience from tertiary care hospital In India. J Diabetes Metab Disord. (2023) 22:1459–69. 10.1007/s40200-023-01269-3 37975119 PMC10638341

[13] Zhang R Jin G Zhan Y Shen L Yao Y Gao Q. Allergic bronchopulmonary mycosis caused by mucor overlapping with invasive pulmonary mucormycosis: a case report. Front Med. (2022) 9:831213. 10.3389/fmed.2022.831213 35280885 PMC8907707

